# Optical Manipulation of Lanthanide-Doped Nanoparticles: How to Overcome Their Limitations

**DOI:** 10.3389/fchem.2020.593398

**Published:** 2020-11-09

**Authors:** Elisa Ortiz-Rivero, Lucía Labrador-Páez, Paloma Rodríguez-Sevilla, Patricia Haro-González

**Affiliations:** ^1^Fluorescence Imaging Group, Departamento de Física de Materiales, Universidad Autónoma de Madrid, Madrid, Spain; ^2^Department of Applied Physics, Royal Institute of Technology (KTH), Stockholm, Sweden; ^3^Scottish Universities Physics Alliance (SUPA), School of Physics and Astronomy, University of St Andrews, St Andrews, United Kingdom; ^4^Instituto Nicolás Cabrera, Universidad Autónoma de Madrid, Madrid, Spain

**Keywords:** nanoparticle, lanthanide, rare-earth, optical trapping, optical force

## Abstract

Since Ashkin's pioneering work, optical tweezers have become an essential tool to immobilize and manipulate microscale and nanoscale objects. The use of optical tweezers is key for a variety of applications, including single-molecule spectroscopy, colloidal dynamics, tailored particle assembly, protein isolation, high-resolution surface studies, controlled investigation of biological processes, and surface-enhanced spectroscopy. In recent years, optical trapping of individual sub-100-nm objects has got the attention of the scientific community. In particular, the three-dimensional manipulation of single lanthanide-doped luminescent nanoparticles is of great interest due to the sensitivity of their luminescent properties to environmental conditions. Nevertheless, it is really challenging to trap and manipulate single lanthanide-doped nanoparticles due to the weak optical forces achieved with conventional optical trapping strategies. This limitation is caused, firstly, by the diffraction limit in the focusing of the trapping light and, secondly, by the Brownian motion of the trapped object. In this work, we summarize recent experimental approaches to increase the optical forces in the manipulation of lanthanide-doped nanoparticles, focusing our attention on their surface modification and providing a critical review of the state of the art and future prospects.

## Introduction

Optical trapping (OT) of nanoparticles (NPs) by the forces exerted by a tightly focused laser beam has allowed innumerable advances in the study of single molecules and cells and the characterization of photonic nanomaterials. OT stands out for its ability to isolate and manipulate NPs for their study in a contactless and non-invasive way, and it has enabled the development of different techniques, such as photonic force microscopy, the optical manipulation for assembly or actuation in nanostructures, and diverse types of local contactless sensing (Maragò et al., 2013; Rodríguez-Sevilla et al., 2017a; Bradac, 2018; Bunea and Glückstad, 2019). For the application of optical tweezers in biological science, Arthur Ashkin, the inventor of the technique, was awarded with the Nobel Prize in Physics in 2018.

The most commonly optically trapped inorganic particles are silica and polystyrene beads. They are usually tracked to infer properties of the environment or employed as handlers for non-direct manipulation of, for example, biological molecules. However, their detection can become challenging if their size is reduced below the resolution limit of the optical setup. In contrast, OT of luminescent particles has the advantage of enabling their detection by their luminescence even if they are smaller than the resolution limit of the imaging system. Moreover, they have a great potential for sensing as most of them show environment-sensitive luminescence. Several types of luminescent particles have been already optically trapped: quantum dots (Jauffred et al., 2008; Jensen et al., 2016), nanodiamonds (Geiselmann et al., 2013; Neukirch et al., 2013, 2015), semiconductor nanowires (Reece et al., 2009; Wang et al., 2011a), niobate nanowires (Nakayama et al., 2007; Dutto et al., 2011), and lanthanide-doped nanoparticles (NPs:Ln) (Haro-Gonzalez et al., 2013; Rodríguez-Sevilla et al., 2015, 2016a; Kang et al., 2019). NPs:Ln stand out due to their photostability, long luminescence decay time, narrow and multiple emission bands, and tailorable luminescence characteristics. Within the NPs:Ln library, upconverting nanoparticles (UCNPs) are the most frequently optically trapped particles because they show low biotoxicity and present biocompatible excitation routes. They can absorb light of low energy and emit photons of higher energy through a non-linear process, e.g., emitting visible light after the successive absorption of two or more infrared photons (Labrador-Páez et al., 2018; Zheng et al., 2019). Thus, the combination of infrared biocompatible laser wavelengths with visible luminescence facilitates the detection and tracking of the trapped particle, making them great candidates for bio-applications as contactless local probes.

Multiple applications of NPs:Ln have been explored. Their temperature-sensitive luminescence can be used for local non-invasive temperature sensing of cells (Rodríguez-Sevilla et al., 2016; Drobczynski et al., 2017). Moreover, their polarized emission allows the measurement of the viscosity of the cytoplasm and the detection of single cells (Rodríguez-Sevilla et al., 2016b; Ortiz-Rivero et al., 2019). Other applications include the measurement of the size of bacteria and the labeling of RNA and cancer biomarkers for their quantification in bioassays (Li et al., 2017, 2018; Xin et al., 2017).

For all the aforementioned outstanding characteristics and the multitude of applications as remote contactless biosensors, the use of NPs:Ln for OT has been growing in the last decade. For these reasons, we have chosen to focus our attention on the OT of NPs:Ln and the challenges that the community is currently facing. In the next sections, we will firstly introduce the fundamentals of OT and its limitations for single NP:Ln manipulation. Then, we will discuss how these can be overcome, and we will envision future fields of application of OT of NPs:Ln.

## Optical Trapping: Fundamentals

Optical manipulation is based on the optical forces that a tightly focused laser beam can exert on a particle thanks to the interaction between the electromagnetic field of the light and matter. The magnitude of these optical forces depends on the properties of the light (power, polarization, and beam shape), but predominantly on the characteristics of the particle (size, shape, and polarizability) (Ashkin et al., 1986; Rohrbach and Stelzer, 2001; Bendix et al., 2014).

From a simplified point of view, the optical force exerted on a particle could be visualized as the combination of the gradient and scattering forces. The gradient force acts as a restoring force that attracts the particle toward the maximum intensity of the laser beam, while the scattering force points in the same direction as the beam propagates and destabilizes the trap, pushing the particle away from the stable position. These forces should be balanced for achieving stable trapping conditions.

In the case of particles much smaller than the wavelength of the trapping light (Rayleigh regime), the scattering force can be neglected and the dynamics of the trapped particle can be described, taking into account only the action of the gradient force (Spesyvtseva and Dholakia, 2016). This is only completely true for nanoparticles with diameters smaller than some tens of nanometers. Bigger nanoparticles are still pushed by the optical scattering force, although its effect is usually neglected for simplification, as NP stable trapping can only be achieved when the gradient force overcomes the scattering force. The gradient force acting on the NP can be expressed as

(1) $$\begin{array}{r} {\text{F}}_{\text{g}\text{ra}\text{d}}=\frac{1}{2}\alpha_{\text{N}\text{P}}\nabla \langle {{\text{E}}_{\text{T}\text{RA}\text{P}}}^{2}\rangle , \end{array}$$

where *E*_*TRAP*_ is the electric field of the trapping light and **α**_**NP**_ is the polarizability of the particle

(2) $$\begin{array}{r} \alpha_{\text{N}\text{P}}=4\pi \varepsilon_{0}{\text{V}}_{\text{N}\text{P}}\frac{\varepsilon_{\text{N}\text{P}}-\varepsilon_{\text{m}}}{\varepsilon_{\text{N}\text{P}}+2\varepsilon_{\text{m}}}, \end{array}$$

which depends on the volume of the particle (*V*_*NP*_), and the electric permittivity of vacuum (ε_0_), the surrounding medium (ε_*m*_), and the nanoparticle (ε_*NP*_). Thus, in this situation, the optical force decreases as the volume of the particle does. This is the main limitation in OT of NPs.

For biological applications, NPs are manipulated in liquid media. Thus, the trapped particle is subjected to the temperature-dependent Brownian fluctuations which destabilize it and make it oscillate around the equilibrium position. If the optical force is not large enough to compensate this motion (i.e., if the optical potential does not exceed at least 10 times the thermal energy *k*_*B*_*T*, where *k*_*B*_ is the Boltzmann constant and *T*, the temperature), the particle will escape the optical trap, making its manipulation impossible.

For this reason, optical forces should be enhanced enabling the manipulation of NPs in liquid media. From expression (1), the magnitude of the optical force depends on the power of the trapping beam (i.e., $F_{grad}\propto {E_{TRAP}}^{2}$). However, increasing the trapping power in favor of higher optical forces would present side effects related to the absorption of the trapping radiation by either the particle or the medium. This could lead to a deterioration of the sample, but more importantly, it can cause an increase in temperature that would enhance the Brownian fluctuations (Peterman et al., 2003; Rodríguez-Sevilla et al., 2017b). Therefore, this solution is usually ruled out.

Other properties of the beam can also be tailored for the enhancement of the optical force. Expression (1) shows that the force depends on the gradient of the intensity of the beam (i.e., $F_{grad}\propto \nabla \langle {E_{TRAP}}^{2}\rangle$). For this reason, far-field optical manipulation of small particles makes use of high numerical aperture objective lenses to focus the laser beam to a small spot comparable to the particle size (Bartlett and Henderson, 2002; Rohrbach, 2005). However, the diffraction limit ($\sim \frac{\lambda }{2\text{NA}}$) restricts the smallest spot that can be generated for a given trapping wavelength (λ) and numerical aperture (NA). For this reason, novel trapping strategies have been developed for OT of entities much smaller than the trapping wavelength (see section Optical Force Increase Based on the Reduction of the Optical Trap Volume).

The properties of the particle can also be tailored to enhance the optical force. The polarizability of the particle depends on the material of which it is made but also on the molecules that surround it (see section Optical Force Increase Based on Surface Modifications). In addition, it depends on the nanoparticle morphology. Anisotropic nanoparticles may show a certain degree of polarization. Their effective induced polarization is non-isotropic, which produced an optical torque to align the electric field with the particle polarization. Thus, the stable orientation inside the optical trap is strongly dependent on the incident electric field and the NP geometrical axis.

The trapped particle can add new functionalities to the optical manipulation tool, since it not only is useful to control its position and motion but also can be used as a force transducer. For this purpose, the optical trap should be calibrated. This can be done by different techniques that are based on the analysis of the motion of the trapped particle (Sarshar et al., 2014). These dynamics can be detected imaging the particle (video-tracking) or from the intensity fluctuation produced in the laser beam when it is scattered by the particle (Bui et al., 2018). The luminescence of NPs:Ln is advantageous since particles much smaller than the diffraction limit can be detected. However, the emission should be intense enough for a good signal-to-noise ratio. This is hard to achieve for very small particles due to their weak emission and the short acquisition times (high frame rates) required to effectively detect the particle dynamics. Although techniques have been developed for the use of limited frame rate (Wong and Halvorsen, 2006), NPs:Ln facilitate the use of this method thanks to their outstanding resistance to photobleaching that permit, for example, to track the particle for long periods of time. Single NP:Ln luminescence has been reported by different groups that managed to achieve the emission spectra of individual trapped nanoparticles based on drop-casting a diluted suspension (Schietinger et al., 2010; Gargas et al., 2014). However, for most bio-applications, the interest falls on assessing single NP emission in a colloidal suspension (Haro-Gonzalez et al., 2013; Rodríguez-Sevilla et al., 2015). The luminescence of single NP:Ln in solution has been spectroscopically characterized, which enabled the comparisons of the performance between different particles, and to study inter-particle interactions (Roder et al., 2015; Zhou et al., 2020).

## Optical Trapping of Lanthanide-Doped Nanoparticles

As explained in section Optical Trapping: Fundamentals, it is challenging to manipulate NPs with conventional OT strategies as the optical forces decrease with the particle volume. In this section, we describe different strategies used to increase the magnitude of the optical forces, which either modify the particle surface (section Optical Force Increase Based on Surface Modifications) or reduce the optical trap volume (section Optical Force Increase Based on the Reduction of the Optical Trap Volume). Table 1 summarizes the most relevant NPs:Ln used to date, as well as the maximum optical forces achieved.

**Table 1:** Classification of the most relevant techniques used on NPs:Ln to enhance their optical force.

**References**	**Nanoparticle**	**Size (nm)**	**Synthesis method**	**Max. trap efficiency (pN μm^**−1**^ W^**−1**^)**	**Strategy used**
**Synthesis/surface modification**					
Anbharasi et al. (2020)	LiYF_4_:Yb,Er	238	Hydrothermal	0.0055	Calcination
Rodríguez-Rodríguez et al. (2015) and Rodríguez-Sevilla et al. (2015)	SrF_2_:Yb,Egr	8	Hydrothermal	0.33	Surface charge tailoring
Kang et al. (2019)	NaYF_4_:Yb, Er	370	Thermal decomposition	1	Hydrophobic encapsulation and ligand exchange
Rodríguez-Sevilla et al. (2018)	NaYF_4_:Yb,Er	8–200	Thermal decomposition	25	Core–shell
**Reduction of the optical trap volume**					
Mor et al. (2014)	NaYF_4_:Yb,Er	230	Flame-fusion and hydrothermal		PFM
Schietinger et al. (2010)	NaYF_4_:Yb,Er	30	Microwave-assisted		AFM tip
Xin et al. (2017)	KLu_2_F_7_:Yb,Er	120	Hydrothermal	14.1	Fiber tip
Leménager et al. (2018)	NaYF_4_:Gd,Yb,Er	600–2,000	Solvothermal	4	Dual fiber tweezers
Kumar et al. (2020)	NaYF_4_:Eu	l:220 0, d:120	Solvothermal	6	Dual fiber tweezers
Li Y. et al. (2017)	NaYF_4_:Yb,Tm @ SiO_2_-NH_2_	28	Hydrothermal	448 (trapped *E-coli* covered with NPs)	Fiber tip + bio-microlens

*They are divided depending on the strategy used: synthesis/surface modification of the nanoparticle or reduction of the optical trap volume. Where “l” and “d” refers to lenght and diameter, respectively. PFM, photonic force microscopy*.

### Optical Force Increase Based on Surface Modifications

OT of NPs:Ln is challenging because they are dielectric particles with low polarizability (α_*NP*_) and the trapping force scale with α_*NP*_. Expression (2) shows that α_*NP*_ depends on the dielectric constants of the NP and that of the environment, so that α_*NP*_ optimization can be achieved by modifying the NP material and/or the surrounding medium. A large number of methods for surface modification of NPs have been reported (Wang et al., 2011b; Hirsch, 2020), which enhance the NPs:Ln luminescence (i.e., easing the tracking) and their colloidal stability, while providing the possibility of subsequent bioconjugation. However, despite its interest, the optimization of the optical forces acting on single NP:Ln through its surface modification is a route that has not been thoroughly explored. Some synthesis/surface modification strategies are included in Table 1.

**Figure 1:**
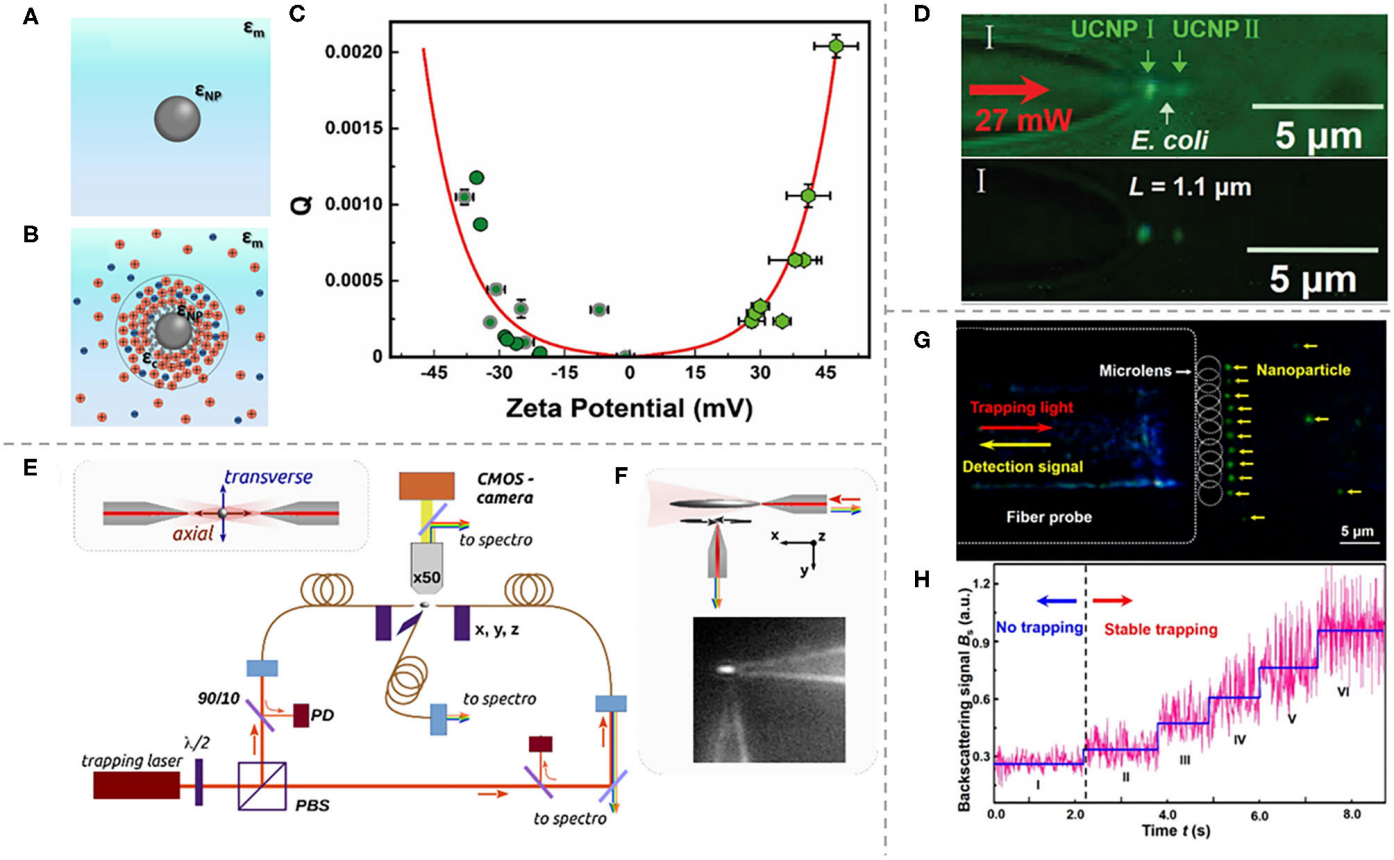
Optical trapping of a NP:Ln in a solvent by different systems based on the reduction of the optical trap volume. **(A)** A ligand-free lanthanide-doped dielectric NP (permittivity ε_NP_) in a medium (permittivity ε_m_). **(B)** A dielectric NP:Ln coated with ligands (coating permittivity ε_c_). The electric double layer is indicated by the circles. **(C)** Optical trapping strength, represented by the Q-factor, as a function of the zeta potential for different types of NP:Ln. Adapted with permission from Rodríguez-Sevilla et al. (2018). Copyright 2018 American Chemical Society. **(D)** Bright-field (top) and dark-field (bottom) images of two NPs:Ln (I and II) and an *E. coli* bacterium trapped in a row by optical fiber tweezers. Adapted with permission from Xin et al. (2017). Copyright 2017 John Wiley and Sons. **(E)** Schematic of a dual optical fiber tweezer system. **(F)** Schematic and microscopy image of a single fiber tip trap including the auxiliary fiber tip used for particle emission recording of a single NP:Ln. OSA Open Access License for OSA-Formatted Journal Article PDFs (Leménager et al., 2018). **(G)** Tip of an optical fiber with an array of coupled spherical microlenses that are able to trap nanoparticles. The fiber probe also records the detection signal. The sequential growth, in backscattering signal, as different nanoparticles are trapped by the microlenses is shown in **(H)**. Adapted with permission from Li Y. et al. (2016). Copyright 2016 American Chemical Society.

Colloidal NPs present a superficial charge which interacts with the solvent's ions. These changes distribute around the particle forming an electric double layer characterized by the zeta potential, as depicted in Figures 1A,B, which influences α_*NP*_. The NPs may have a surface coating to improve their colloidal properties and zeta potential. For the optimization of α_*NP*_, Rodríguez-Rodríguez et al. (2015) studied the influence of the surface coating of 8-nm SrF_2_:Er,Yb NPs:Ln on the trapping efficiency. They increased α_*NP*_ by replacing the cationic species on the NP surface by more mobile ones in solution. This modification led to an almost 50-fold enhancement in the trapping efficiency, showing that the contribution of the surface coating to the net polarizability dominates over that of the NP:Ln core material. This study was continued by Rodríguez-Sevilla et al. (2018), determining the optical forces acting on NaYF_4_:Er,Yb NPs:Ln of different sizes (ranging from 5 to 100 nm). They experimentally demonstrated that the optical forces (parameterized by the trapping factor Q) acting on a NP:Ln depend on the electrostatic properties (zeta potential) of the interface between the NP and the surrounding medium more strongly than on their volume (see expression 2 and Figure 1C). Alternatively, selecting the right medium (the solvent molecules) can be essential to optimizing the zeta potential value. Likewise, the temperature of the medium, which affects its permittivity (Catenaccio et al., 2003) and conductivity (Cao et al., 2019) and indirectly α_*NP*_, can lead to the enhancement of the optical forces, though the Brownian motion increase may be detrimental.

### Optical Force Increase Based on the Reduction of the Optical Trap Volume

An alternative strategy to improve the optical forces acting on single NP:Ln consists of the reduction of the optical trap size to increase the electric field gradient [see expression (1)]. When the dimensions of a bulk material are reduced, new properties appear such as the ability to confine light into a region much smaller than the wavelength (Koya et al., 2020). With that purpose, plasmonic effects and optical resonances have been employed to enhance the trapping forces and luminescence of NPs:Ln.

Optical nanotweezers are metallic or dielectric nanostructures that can generate strong electromagnetic field gradients as they can confine the light to a subwavelength region using lower laser powers than conventional OT (Shoji and Tsuboi, 2014; Huang and Yang, 2015). Their development was mainly motivated by the aim to suppress the scattering forces for NP trapping (Min et al., 2013).

Nanotweezers have been proven to allow the manipulation and detection of single NP and small molecules, controlling their motion in the nanoscale. Different plasmonic and dielectric nanostructures have been developed for OT of luminescent NPs (e.g., polystyrene fluorescent beads or quantum dots Yoo et al., 2018; Xu and Crozier, 2019; Kotsifaki et al., 2020). Furthermore, the luminescence of the trapped particle can be excited by an additional beam or through an upconversion process using the trapping beam. Although the manipulation of sub-30-nm dielectric nanoparticles by plasmonic optical tweezers has been demonstrated, the manipulation of NPs:Ln by this technique is yet to be achieved (to our knowledge), which would bring the outstanding capabilities of these particles to the nanoscale.

In a different strategy, several studies have developed innovative optical tweezers based on optical fibers, which was firstly demonstrated by Fuh et al. (1987) and Constable et al. (1993). As an alternative to bulky high-numerical aperture objective lenses, optical fibers can create a sub-diffraction spot at their tip if it is judiciously modified, i.e., providing it with a lenticular shape (Li et al., 2015; Li Y.-C. et al., 2016; Li Y. et al., 2017). This has the advantage of compactness and high manipulation flexibility due to their reduced size. Moreover, it also results in different trapping and luminescence recording configurations. The focusing capabilities of the optical fiber can be optimized by modifying the shape of its tip and, for example, overcome the diffraction limit (Berthelot et al., 2014; Asadollahbaik et al., 2020; Zhao et al., 2020). Direct trapping of NPs:Ln has been achieved with a single fiber-coupled laser, reducing drastically the size of the experimental setup. H. Xin et al. used a tapered fiber tip capable of tightly focusing the output light resulting in a high-intensity gradient. As shown in Figure 1D, they employed this strategy to measure the length of single bacterium by co-trapping in a row a KLu_2_F_7_:Yb,Er NP:Ln of 120 nm (with a trap stiffness of 14.1 pN/m W), a single *E. coli* bacterium, and then a second NP:Ln (12.8 pN/mW). In comparison with other Yb, Er co-doped NPs:Ln included in Table 1, their trapping stiffness was several orders of magnitude larger, demonstrating the efficiency of this approach.

Another approach to optimize OT of NPs is based on dual fiber tweezers. Counter-propagating traps based on two opposite fibers have been proven to be able to stably trap NPs:Ln for the study of their luminescence. Although the optical forces exerted on the NPs have not been reported, their magnitude is expected to be higher than for conventional OT. For example, Leménager et al. (2018) recorded the anisotropic emission of trapped NaYF_4_:Er,Yb,Gd nanorods in three orthogonal directions using distinct methods: through the microscope objective, by coupling it into one of the trapping fiber tip, and by coupling it into a third fiber (see Figures 1E,F). In a similar way, Kumar et al. (2020) studied nanorods of NaYF_4_:Eu, measuring their 3D orientation by europium ion polarization-dependent luminescence.

The dual-beam configuration can be further improved by its combination with the modification of the fiber tip, adding a micro-lens. The main advantage of using microscopic lenses over typically macroscopic ones is their considerably smaller focusing spot size and mobility inside the sample. This has been reported to enhance the optical forces for nanosized polystyrene particles so it could also be used for NP:Ln. For instance, Asadollahbaik et al. positioned 3D-printed diffractive Fresnel lenses at the tip of the fibers in a counter-propagating arrangement. This novel miniaturized optical setup can produce a variety of NA with a large working distance and a reduced trapping spot, increasing the trap stiffness by a factor of 35–50 (1762.87 pN/μm W). On the other hand, a microlens can also be realized by a colloidal particle such as a dielectric microcylinder or microsphere.

When it is optically trapped, the particle focuses the light and a nanosized photonic jet is generated at its shadow-side surface. Photonic nanojets can propagate over a distance corresponding to several optical wavelengths without significant divergence, and their waist size is below the diffraction limit, depending on the microlens diameter, diffraction index, and laser wavelength (Chen et al., 2004; Neves, 2015). In addition, the light backscattering and the nanoparticle luminescence can be enhanced and recorded, making fiber-coupled microspheres suitable for NPs:Ln optical manipulation. Li et al. reported different types of cells as natural bio-microlens. Then, NPs:Ln were coupled to pathogenic bacteria, which then were subsequently trapped individually or in chains. The maximum trapping efficiency they obtained was 448 pN μm^−1^ W^−1^ for an NP:Ln-covered *E. coli* bacterium. More complex systems present a parallel photonic nanojet array, produced by assembling and binding microlenses on a single optical fiber tip, as depicted in Figures 1G,H (Li Y. et al., 2016; Schäffner et al., 2020).

## Future Perspectives and Conclusions

Lanthanide-doped nanoparticles present unique, environment-sensitive, selective, and bio-compatible spectroscopic characteristics that stand out among other dielectric luminescent nanoparticles. Hence, they are ideal for optical trapping, a non-invasive and versatile tool used to manipulate small objects. Their main drawback is their reduced size of nanoparticles, which is below the diffraction limit of the optics system and reduces the achievable optical forces. In this review, we have analyzed the strategies that have been developed or would be of potential use to overcome this limitation, i.e., the modification of the lanthanide-doped nanoparticle's characteristics and the optimization of the optical tweezer setup. Some of these techniques can also enhance the luminescence of the lanthanide-doped nanoparticle, which is of great interest for numerous applications. Despite the potential of the reviewed techniques, their implementation is an underdeveloped field. We think these already proven strategies could open the door to widen the application of lanthanide-doped nanoparticles, which present better capabilities and multifunctionalities than other commonly used nanoparticles.

## Author Contributions

All authors listed have made a substantial, direct and intellectual contribution to the work, and approved it for publication.

## Conflict of Interest

The authors declare that the research was conducted in the absence of any commercial or financial relationships that could be construed as a potential conflict of interest.
